# Supplementary material for: Mouse embryonic stem cells can differentiate via multiple paths to the same state
Source: eLife. 2017 Oct 9;6:e26945. doi: 10.7554/eLife.26945 (PMC5648529; doi:10.7554/eLife.26945)
Supplement: Transparent reporting form [file elife-26945-transrepform.pdf]

eLife's transparent reporting form

We encourage authors to provide detailed information *within their submission* to facilitate the interpretation and replication of experiments. If you have any questions, please contact us: editorial@elifesciences.org.

Sample-size estimation

- You should state whether an appropriate sample size was computed when the study was being designed
- You should state the statistical method of sample size computation and any required assumptions
- If no explicit power analysis was used, you should describe how you decided what sample (replicate) size (number) to use

Please outline where this information can be found within the submission (e.g., page numbers or figure legends), or explain why this information doesn't apply to your submission:

Sample sizes for single cell experiments were selected to allow robust detection of rare populations and are indicated on pages 2, 9, and in the supplemental methods. Sample sizes for all other experiments are described in the supplemental methods.

Replicates

- You should report how often each experiment was performed
- You should include a definition of biological versus technical replication
- The data obtained should be provided and sufficient information should be provided to indicate the number of independent biological and/or technical replicates
- If you encountered any outliers, you should describe how these were handled
- Criteria for exclusion/inclusion of data should be clearly stated
- High-throughput sequence data should be uploaded before submission, with a private link for reviewers provided (these are available from both GEO and ArrayExpress)

Please outline where this information can be found within the submission (e.g., page numbers or figure legends), or explain why this information doesn't apply to your submission:

Combining the single-cell and validation experiments, the two differentiation protocols were each performed >6 times (each being an independent biological replicate). Numbers of technical replicates, and details on data filtering criteria are indicated on pages 2, 9, and in the supplemental methods. Private GEO links to raw and processed data are provided in the online submission metadata.

eLIFE
1st Floor
24 Hills Road
Cambridge CB2 1JP, UK
P 01223 855340
W elifesciences.org
T @elife

Statistical reporting

- Statistical analysis methods should be described and justified
- Raw data should be presented in figures whenever informative to do so (typically when N per group is less than 10)
- For each experiment, you should identify the statistical tests used, exact values of N, definitions of center, methods of multiple test correction, and dispersion and precision measures (e.g., mean, median, SD, SEM, confidence intervals; and, for the major substantive results, a measure of effect size (e.g., Pearson's r, Cohen's d)
- Report exact p-values wherever possible alongside the summary statistics and 95% confidence intervals. These should be reported for all key questions and not only when the p-value is less than 0.05.

Please outline where this information can be found within the submission (e.g., page numbers or figure legends), or explain why this information doesn't apply to your submission:

Statistical tests used are named in the main text and figure legends throughout the manuscript, along with their p-values reported to >3 decimal places. Details on their implementation are provided in the supplemental methods. Raw data is provided through GEO and in Figure 2-figure supplements 1+2, Figure 4-figure supplement 1, and Figure 5-figure supplement 1.

(For large datasets, or papers with a very large number of statistical tests, you may upload a single table file with tests, Ns, etc., with reference to page numbers in the manuscript.)

Additional data files ("source data")

- We encourage you to upload relevant additional data files, such as numerical data that are represented as a graph in a figure, or as a summary table
- Where provided, these should be in the most useful format, and they can be uploaded as "Source data" files linked to a main figure or table
- Include model definition files including the full list of parameters used
- Include code used for data analysis (e.g., R, MatLab)
- Avoid stating that data files are "available upon request"

Please indicate the figures or tables for which source data files have been provided:

Single-cell RNA sequencing (Figs. 2-5) and microarray data (Fig. 4) are available in raw and processed data formats through GEO. Private reviewer links are provided to these files in the online submission metadata. Major pieces of code previously developed in the Klein lab (including SPRING, PCA-tSNE, and low-level bioinformatics pipelines) are referenced in text and publically available.

eLife Sciences Publications, Ltd is a limited liability non-profit non-stock corporation incorporated in the State of Delaware, USA, with company number 5030732, and is registered in the UK with company number FC030576 and branch number BR015634 at the address 1st Floor, 24 Hills Road, Cambridge CB2 1JP | August 2014
